# Quantitative 3D analysis of complex single border cell behaviors in coordinated collective cell migration

**DOI:** 10.1038/ncomms14905

**Published:** 2017-04-04

**Authors:** Adam Cliffe, David P. Doupé, HsinHo Sung, Isaac Kok Hwee Lim, Kok Haur Ong, Li Cheng, Weimiao Yu

**Affiliations:** 1 Institute of Molecule and Cell Biology (IMCB), Computational Bioimage Analysis Unit, A*STAR, Singapore 138673, Singapore; 2 Bioinformatics Institute (BII), Imaging Informatics Division, A*STAR, Singapore 138671, Singapore

## Abstract

Understanding the mechanisms of collective cell migration is crucial for cancer metastasis, wound healing and many developmental processes. Imaging a migrating cluster *in vivo* is feasible, but the quantification of individual cell behaviours remains challenging. We have developed an image analysis toolkit, CCMToolKit, to quantify the *Drosophila* border cell system. In addition to chaotic motion, previous studies reported that the migrating cells are able to migrate in a highly coordinated pattern. We quantify the rotating and running migration modes in 3D while also observing a range of intermediate behaviours. Running mode is driven by cluster external protrusions. Rotating mode is associated with cluster internal cell extensions that could not be easily characterized. Although the cluster moves slower while rotating, individual cells retain their mobility and are in fact slightly more active than in running mode. We also show that individual cells may exchange positions during migration.

## Abstract (web summary)

Quantifying cell behaviours *in vivo* is essential to understanding the mechanisms of collective cell migration. Here the authors present an image analysis toolkit, CCMToolKit, to describe and characterize various modes of coordinated cell movements accompanying collective cell migration in *Drosophila* border cells.

Different types of cells in various contexts migrate together as groups rather than as isolated entities^1^,^2^. This collective migration of multiple cells is highly directed and coordinated. It is a highly dynamic process involved in immune response, wound healing, tissue development, and cancer metastasis. Many studies of collective cell migration have been undertaken in two-dimensional (2D) tissue culture^3^. Although 2D experiments have provided many insights into general principles, the situation is very different from the endogenous three-dimensional (3D) environment. It has been reported that *in vivo* migration behaviour significantly differs from movement on hard 2D substrates^4^,^5^. To study cells in a 3D context, we can either make *in vitro* substrates similar to natural conditions or observe collective cell migration directly in the tissue. 3D *in vivo* experiments are the most physiologically relevant but demand the optimization of imaging protocols and advanced image analysis methods.

For *in vivo* studies, 3D time-lapse imaging is becoming less problematic due to advances in fluorescent labelling and microscopy. However, after 3D time-lapses are acquired, a challenging step is to analyse those image stacks using computational approaches to extract meaningful data. The quantitative 3-D analysis should be conducted on relatively large data sets covering multiple movies/cells and extended periods of observation since the biological variation of both the migratory clusters and substrate composition/geometry should be considered. To achieve this requires an automatic, efficient and accurate computational solution to extract relevant quantitative information to better understand the complex behaviours of both the individual cells and the cluster as a whole.

In this paper, we focus on the well-established model of border cells migrating in the *Drosophila* ovary^6^. The migrating cells form a closely packed cluster, comprised of a pair of non-motile hemispheric polar cells, which are surrounded by border cells. The border cells detach from the follicular epithelium and then migrate together between large nurse cells towards the oocyte. The complete process is highly reproducible and takes about 3–4 h. The process is guided by some diffusible cues generated by the oocyte, signaling through the receptor tyrosine kinases PDGF/VEGF receptor (PVR) and epidermal growth factor receptor^7^,^8^,^9^. Previous studies have successfully imaged this process in live isolated egg chambers^7^,^10^. It requires the homophilic adhesion molecule DE-cadherin on both the border cells and substrate nurse cells^11^. The cytoskeletal regulator RAC also plays a critical role in guiding migration of the border cell cluster^12^. A more recent work suggested a positive mechanical feedback model for such guidance^13^.

In some previous studies, 3D imaging was performed, however, for simplicity, the image data was analysed and interpreted in 2D, either based on single sections or 2D projections of image stacks. Morphodynamics analysis has revealed different patterns of cell extension. However it is still limited to 2D and based on the 2D outer contour of the whole cluster, rather than individual cell in 3D^13^. Overall, this migration process is highly directed, but it contains both chaotic and coordinated movements. Compared to chaotic movement, the coordinated behaviour is more important and relevant to the directed migration. During the coordinated migration, all border cells have similar behaviours, either moving in the same direction (running) or rotating along a given axis (rotating). In general, such coordinated collective movement of cells as a multicellular unit is not fully understood in many biological processes. Furthermore, we do not have the means to quantify these different motions in 3D. At the single cell level, although we know cells display complex behaviours characterized by the cyclic protrusion, adhesion, and contraction of processes in 3D^11^,^12^,^13^, our understanding of the 3D morphology dynamics of every individual cell is limited due to a lack of suitable computational methods. Here we have studied each cell movement and their morphology within the cluster, providing insights into the mechanisms of coordinated cell movement driving the migration of the cluster as a unit.

Computational solutions have been reported to address collective cell migration^14^,^15^,^16^,^17^, but they are difficult to apply to the border cell system. The challenges of implementing those solutions include the image quality, tracking objects with highly variable speed, and segmentation of the cell surfaces based on weak membrane signal. Overall, a systematic solution designed for this purpose would be extremely useful. For example, a recent development of the ADAPT^18^ ImageJ plug-in can analyse cell morphology changes and detect protrusions and blebs, but is again limited to 2D and lacks the capability to separate closely packed cells. In summary, a computational solution to accurately and automatically segment, track and quantitatively describe the complex behaviour of every individual polar/border cell and the cluster in 3D over time is currently lacking.

Here we present an automatic computational method of 3D reconstruction, segmentation, and tracking, enabling the quantitative analysis of migrating border cells on a cell-by-cell basis. Our solution, the Collective Cell Migration Toolkit (CCMToolKit), is freely available at https://sites.google.com/site/ccmtoolkit/. Quantitative information, such as speed, volume, morphology and movement trajectories of each nucleus and cell, are extracted. The obtained per-cell based information provides us with an extensive detail on how the cells are coordinated together and move forward. In addition to the conventional parameters for individual cells' behaviour, we also quantify cell–cell interactions within the cluster, for example, the interacting surface between two border cells and the degree of coordination. We also quantify neighbour exchange as adjacent border cells change their relative positions with each other within the moving cluster.

This numerical solution enables us to address some open questions, many of which are difficult or impossible to characterize in 2D. To quantify different types of coordinated cluster movement, we provide two parameters, Group Polarization (GP) and Angular Momentum (AM), to classify directed and tumbling motions of the cluster, that is, the running and rotating modes, which were qualitatively observed^2^,^5^,^6^,^9^,^11^,^12^,^13^. A numerical classification boundary is provided to differentiate these two modes, but there is a continuum of behaviours between them. We also show that the polar cells usually rotate together with the cluster, but individual border cells can slide along the surface of polar cells in a process we refer to as neighbour exchange. We provide a numerical metric (temporal topology change metric) to detect this neighbour exchange automatically. Clusters generate protrusions during both running and rotating modes, but it has been difficult to quantify their frequency and contribution to the two modes. A computational method is presented to detect cluster protrusions and quantify their role in both running and rotating. Assuming the temporal interval of image acquisition is sufficiently small, we may consider the deformation of the cell surface between two time points as linear. We therefore propose a method to calculate the deformation of each cell according to linear points mapping, offering new insights into cell extension within the cluster and its association with cluster rotation and neighbour exchange. Finally, we address the question of whether individual cells move more slowly during rotating mode.

## Results

### Segmentation and reconstruction of migrating cell cluster

3D time-lapse movies of migrating border cell clusters were generated as described in Methods. We use pre-processing steps to address the problems of photo-bleaching, anisotropic voxel size, poor signal-noise-ratio and the mixture of signals (the fluorescent protein used to label the border cell nuclei has very similar emission spectrum to the nurse cell membrane marker), see Methods section for more details. The image stacks are reconstructed and enhanced at each time point. The photo-bleaching is corrected as shown in Supplementary Fig. 1. We segment the migrating cell nuclei based on the Gaussian Mixture Model (GMM). A dedicated rotational kernel follows this to enhance the weak membrane signal further. To reduce the computational cost, we rotated a kernel in *x*–*y*, *x*–*z*, *y*–*z* planes, convoluted the kernel with membrane signal and enhanced the membrane signal based on the maximum response of the filter bank. A similar membrane enhancement scheme was reported by Mosaliganti *et al*^19^. A marker-controlled watershed approach is then applied to segment the cell membranes.

**Figure 1:**
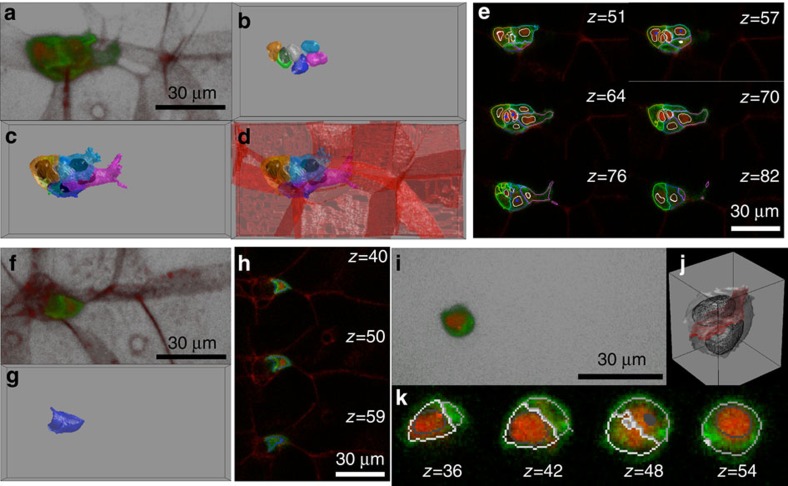
Segmentation and reconstruction of migratory cells and nuclei in the cluster. (**a**) *Z*-projection of an original image stack at a single time point showing migrating cell nuclei (red), membranes (green) and nurse cell membranes (red). (**b**) Segmented nuclei with different cell identities in different colors. Two polar cells are in light and dark grey. (**c**) Reconstructed migrating cell surfaces in 3D with nuclei in black and surface colors as in **b**. (**d**) Reconstructed migrating cells/nuclei and nurse cell surfaces (red). (**e**) Cell boundaries of colors as in **c** and nuclei outlines (white) at different *z*-slices of a cluster in **a**. (**f**) Original image stack of single border cell labelling with border cell membrane (green) and nurse cell membranes (red). (**g**) Reconstructed single border cell surface in blue. (**h**) Slice view of the original signal overlaid with the border cell contour at different *z*-slices. (**i**) Original image stack of polar cell labelling. (**j**) Reconstructed polar nuclei surfaces (meshed surface) and cell surfaces (light and dark grey). Red indicates the interacting surface between two polar cells. (**k**) Slice view of the original signal with the polar cell surfaces (light and dark grey) overlaid at different z-slices.

Our method reconstructs the collective migration of border cells in 4D (3D and time), for both cells and nuclei, with high accuracy and reliability. We resolve the surface of each nucleus, cell and its nurse cell substrates as shown in Fig. 1a–d. We overlaid the cell surface (colour-coded contour) with the original image to visually inspect the segmentation quality, and representative *z*-slices are shown in Fig. 1e. In our segmentation, it is crucial to constrain the cluster volume as a constant, such that the volume of each cell is optimized. To define border/polar cell volume, we acquired movies where only one cell within the cluster is labelled as shown in Fig. 1f,g. The boundaries of a border cell are overlaid with the original image in Fig. 1h. Example data of polar cells are shown in Fig. 1i–k with the interacting surface between the two polar cells visualized in Fig. 1j. These specifically labelled image stacks provide us with cell volume and based on that we constrain the total cluster volume according to the cell number. The boundaries between the cells are determined by the enhanced membrane signal and the marker-controlled watershed algorithm. Accurate segmentation facilitates the nucleus/cell tracking that follows and is a critical step to investigate the behaviour of each cell within the migrating cluster. The 4D reconstruction of a moving cluster, including both nuclei and cell surfaces, are shown in Supplementary Movie 1a–b and a segmentation result at different z-slices is shown in Supplementary Movie 2. For the purpose of validating our computation results, 10 cells (with ∼10–39 time points for each cell) were randomly selected from different movies and manual segmentation was performed. Our computational results are highly consistent with manual segmentation as illustrated in Supplementary Fig. 2. Overall, the true positive rate is >82%, the false negative is about 15% and the false positive is about 30%. A visual comparison shows that false positives are mainly due to manual segmentation failing to captured fine cell structures. Notably, our computational results show much smaller cell volume fluctuations over time than the manual methods in Supplementary Fig. 3 indicating that computational segmentation is more consistent than manual segmentation.

### Tracking nuclei/cells and cell–cell coupling

**Figure 2:**
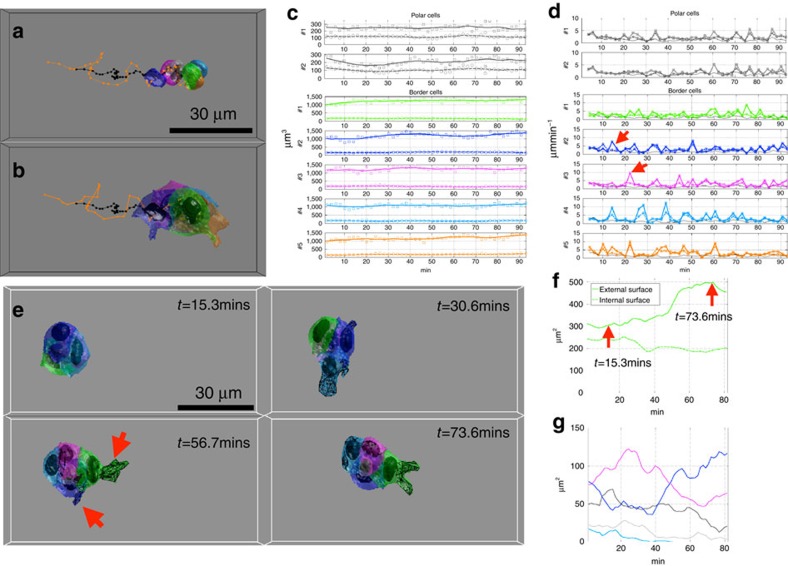
Quantitative tracking of migrating cells and cell-cell coupling. (**a**,**b**) Tracking of migratory nuclei (**a**) and cells' (**b**) mass center. Different colors indicate cell identities. The trajectories in orange are the moving path of the orange cell (**b**) and its nucleus (**a**). Although cluster mass center moves relatively straight and directed (as shown by the black trajectory), each cell does not move as straight as the cluster center and has individual behaviour within the cluster. (**c**) Quantification of nucleus (dashed line) and cell (solid line) volume during migration for each cell. The color code indicates the cell identities as in **a**,**b**. The nuclei and cell volumes are relatively stable. (**d**) Velocity magnitudes of each individual nucleus (dashed line) and cell (solid line). Cells are mostly at low speed (<5 μm min^−1^), but at some time points can be at much higher speed (∼10 μm min^−1^). (**e**) Morphology of the cells and cluster at four different time points. (**f**) Dynamics of internal and external surfaces of the green cell in **e**. Although the internal surface is almost a constant, the external surface increased about 40% at the latter stage of this movie highlighted by the red arrows. (**g**) Cell–cell interacting interface between the green cell and other color-coded cells as in **e**. The green cell initially has more interaction with the magenta cell, while the interacting interface with the blue cell approximately doubles in the latter half of the movie.

The defined tracking cost function in Methods guarantees a global minimum and is able to handle relatively large displacements of nuclei/cells during migration. The tracking analysis provides us with the dynamics of each migrating cell and its nucleus in 4D. The mass center trajectories of one cell and its nucleus are illustrated in Fig. 2a,b, respectively. The cluster mass center trajectory is also shown in black. It is clear that although the cluster movement is relatively straight, an individual cell/nucleus does not move in strict straight line. The volumes of each polar and border nucleus/cell are presented in Fig. 2c. In this panel and Fig. 2d (results of nuclei/cell speed), grey colour indicates two polar cells and other colours are individual border cells as in Fig. 2a,b. As we already knew there is no mass exchange between cells during the migration and border cells do not divide, the volume of each cell is relatively stable (Fig. 2c). By analysing the speed of both nuclei and cells (Fig. 2d), the border cells usually move at low speed (<5 μm min^−1^), but display short bursts of higher speed ∼10 μm min^−1^. This indicates migrating border cells can frequently exhibit individual, uncoordinated intra-cluster motility. The speeds of nuclei are slightly lower than the cells and show less fluctuation (Fig. 2d and Supplementary Fig. 4) but they are highly correlated (Supplementary Fig. 5). In general, polar cell and nucleus speed is lower than that of border cells/nuclei, consistent with a more passive role in migration.

In previous works, the border cell velocity was measured using 2D projections at ∼1.5 μm min^−1^. Here segmentation and tracking individual cells accurately in 3D, allows us to quantify border cell speed in 3D space. The average speed of polar and border cell is about 2–3 μm min^−1^. Our reported value is higher than previous 2D reports because the speed along the z-axis is now taken into account. Indeed, when we remove speed in the z-direction our results show an average speed of 1.7 μm min^−1^, consistent with previous reports. Although the movement of border cells along the *z* axis does not contribute directly to the cluster reaching its destination, it allows us to better understand the overall border cell behaviour in 3D. As polar cells form the center of the cluster and do not touch the nurse cells, their movement is analogous to the net cluster movement (Supplementary Fig. 6).

The reconstruction of each cell's surface in 3D at different time points allows us to look into how the cells are interacting with each other to achieve coordinated movement. Each cell typically has two types of interface: external interface, the surface touching the substrate, that is, the nurse cells; and internal interface, the surface interfacing with other migrating border cells or polar cells.

To calculate the external and internal surface of one given cell, we dilate it by a 3 × 3 × 3 structural element, effectively making that particular cell slightly ‘fatter'. On the basis of the areas that now overlap with other cells, we may then estimate the interacting surface. Although the volume of a border cell is relatively stable, its external and internal interfaces may vary significantly. In Fig. 2f, we show that the green cell in the Fig. 2e cluster has less interaction with nurse cells at the beginning, that is, a smaller external interface, but this contact area increases about 40% at the latter stages of this movie while the internal interface remains approximately constant. The increase in the external interface is closely associated with the generation of a large cluster external protrusion in the second half of this movie. The interface between border cells, which was not accessible, is a key parameter to understand the cell–cell interaction and coordination during the collective movement. The area and variation of these interfaces may indicate the strength of binding between cells and the overall interacting patterns between all cells define the coordination of the cluster. Such analysis allows the study of the interactions between border cells and shows that the cluster internal interaction between cells can change dramatically during the migration (Fig. 2g).

### Running versus rotating modes and polar cell orientation

**Figure 3:**
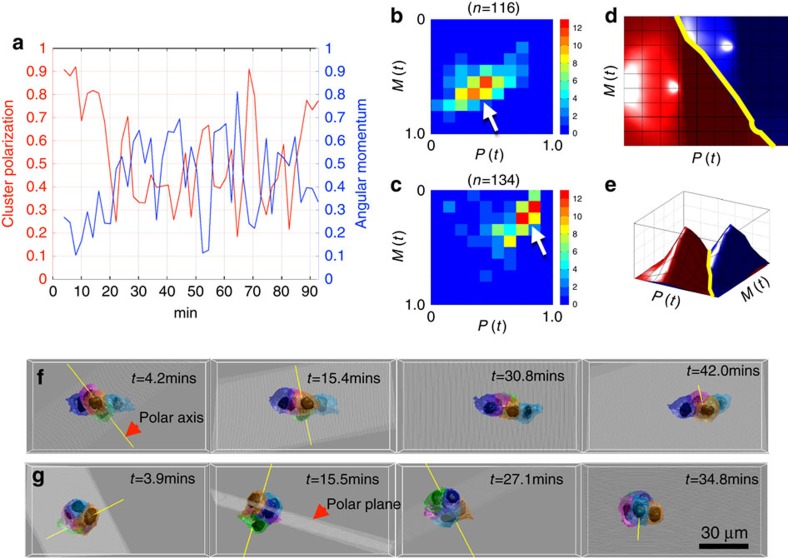
Coordination of migratory cells: running and rotating modes. (**a**) The measurements of group polarization and angular momentum of a movie over time. The values of group polarization and angular momentum are within [0, 1]. We can project to a 2D space and classify the cluster movement into running and rotating. (**b**,**c**) Probability distribution of group polarization and angular momentum of four movies with purely rotating (**b**) or running (**c**). There are 116 time points for cluster rotating and 134 time points for running. In this 2D space, we can clearly see two distinct motion regimes, indicated by white arrows. (**d**,**e**) GMM fitting of group polarization and angular momentum of rotating (red surface) and running (blue surface). The decision boundary of these two modes, indicated by the yellow line, is the edge between the red and blue surface. (**f**) Polar axis (yellow) and plane (light grey) for a running movie. The relative position of the two polar cells is relatively stable during the running mode (Supplementary Movie 5). (**g**) Polar axis and polar plane for a rotating movies. The polar cell rotates with the whole cluster during the rotating mode. (Supplementary Movie 5).

Although the cluster exhibits random and chaotic movements, highly coordinated modes of movement, running and rotating, have been qualitatively observed^2^,^5^,^6^,^9^,^11^,^12^,^13^. However, the mechanism, which governs the coordination between the cells, is not clear and we also lack quantitative parameters to quantify and classify these two different modes. We define two features, group polarization (GP) denoted by *P*(*t*) and angular momentum (AM) denoted by *M*(*t*), to quantify the coordination. Full details of these two parameters are provided in Methods. Briefly *P*(*t*) describes how well the movement of each nucleus/cell is aligned within the cluster. When all cells move in a similar direction, it indicates a high value of *P*(*t*), as can be seen in running mode. Similarly, *M*(*t*) tells us if the cells are rotating in a similar direction. The values of both *P*(*t*) and *M*(*t*) of a movie are illustrated in Fig. 3a.

During certain periods of cluster migration, border cells exhibit highly coordinated movements, as either all cells are moving towards their destination or rotating around the polar cells. We can quantify them as two distinct migration modes based on *P*(*t*) and *M*(*t*). In running mode, *P*(*t*) has higher value, but *M*(*t*) has smaller value; whereas in rotation mode *P*(*t*) has smaller value, but *M*(*t*) has higher value. In Supplementary Movie 3–4, we demonstrate the difference between the running and rotating modes. In these videos, the yellow line is so-called polar axis, which is a straight line passing through the geometric centers of the two polar cells. This allows us to visualize if the polar cell is rotating with the border cell when a cluster is rotating.

We selected 4 movies, which apparently exhibit either only running (134 time points) or rotating (116 time points) modes. We calculate *P*(*t*) and *M*(*t*) and then project their distribution in *P*(*t*) versus *M*(*t*) space for rotating movies (Fig. 3b) and running movies (Fig. 3c). The local maxima in Fig. 3b is shifted in Fig. 3c, indicated by the white arrows. The cluster forward speed difference between running and rotating is shown in Supplementary Fig. 7. Although rotating mode produces little cluster forward speed, cells are still well coordinated within the cluster.

Although we may quantify the coordinated movement as running and rotating modes, we need a general principle to differentiate these two modes and decide whether a cluster at a given time point is under running mode or rotating mode based on these two parameters. We fit our data using a 2D Gaussian model in Fig. 3d,e, where red surface indicates rotating and blue surface indicates running. The yellow boundary in Fig. 3d defines its separating surface between running and rotating modes. Supplementary Fig. 8 shows the distribution of the two features for 42 migrating clusters. The classification of running and rotating mode can be achieved by applying the decision boundary indicated by the yellow line in Fig. 3d and Supplementary Fig. 8. In Supplementary Fig. 8, most of the cluster movements are located at the diagonal of the matrix of *P*(*t*)and *M*(*t*), indicating that overall the cluster are highly coordinated. However, it is clear that there is a continuum of behaviours and some will not fall neatly into either category, that is, clusters with low *P*(*t*)and *M*(*t*). In Fig. 2d, we show that the border cells move at low speed (<5 μm min^−1^) most of the time, but display short bursts of higher speed ∼10 μm min^−1^. These bursts are often not seen in all cells/nuclei in the cluster at a given time (see red arrows in Fig. 2d). This shows that although cells usually move in a highly coordinated fashion as in Supplementary Fig. 8, border cells can still frequently exhibit individual, uncoordinated intra-cluster motility. These intermediate states and the transitions between chaotic and coordinated movement will be of interest for further investigation.

Another open question we can interrogate is whether the polar cells are rotating together with the border cells or remaining in the same orientation while border cells slide over the polar cell surfaces. In Fig. 3f,g, we visualize the polar axis and the polar plane, a plane perpendicular to this axis. We show that the polar axis generally rotates together with the whole cluster during rotating mode in Fig. 3g but is very stable during the running mode. Supplementary Movie 5 clearly demonstrates the difference in polar axis rotation between the running and rotating modes. We have quantified this by taking the moving average of polar axis rotating angles (over 5 time-points) for both running and rotating movies (Supplementary Fig. 9).

The cell–cell interaction surface is an indicator of how the cells are coordinated, and we wondered if it is different in running and rotating modes. Supplementary Fig. 10 shows that the interaction surface is more consistent during running (Supplementary Fig. 10a) than rotating (Supplementary Fig. 10b).

### Neighbour exchange during the cluster migration

**Figure 4:**
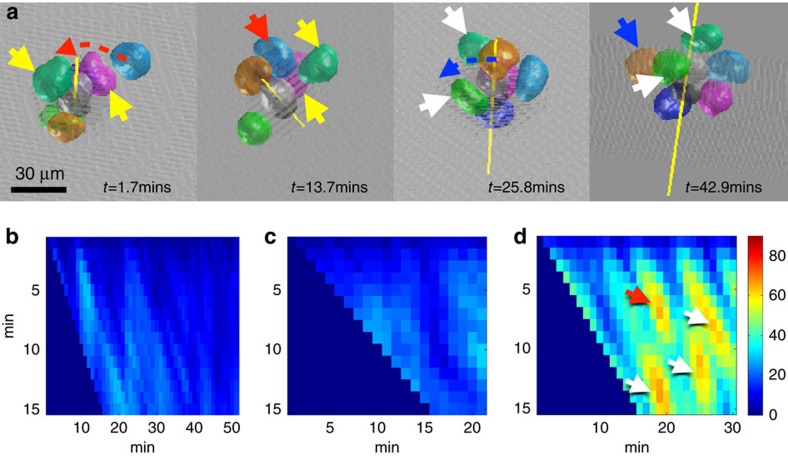
Neighbour exchange and its association of cluster internal cell extensions. (**a**) Two incidences of neighbourhood exchange in a movie. The light blue cell (represented here by its nucleus), whose movement is indicated by the red dotted arrow at *t*=1.7 m, penetrates between the green and magenta cells, indicated by the yellow arrows at *t*=1.7 m and *t*=13.7 m, within ∼10 m. The gold cell, whose path is indicated by the blue dotted arrow, has similar behaviour and moves between the two green cells indicated by the white arrows. (Supplementary Movie 6). (**b**,**c**) The temporal topology change metric of two clusters, running and rotating respectively, without neighbour exchange. The reconstructed movies are shown in Supplementary Movie 7 and 8, respectively. (**d**) The temporal topology change metric of a cluster with four neighbour exchanges. The reconstructed movies are shown in Supplementary Movie 6. The *x* axis of hot peaks indicates the time points of such incidences occurring. The *y* axis indicates how long this neighbour exchange will take in min.

Both the whole cluster behaviours and the individual cell behaviours have been observed to be highly dynamic. A cell may occupy a leading position at one time point, then be found at the rear or side of the cluster later. This process could occur by rotation of the whole cluster as already reported in refs 2, 13. Another scenario is that a cell actively exchanges position with its neighbours, which we term neighbour exchange. Two such examples are shown in Fig. 4a. The light blue cell (represented by its nucleus), moving along the path of the red dotted arrow at *t*=1.7 m, passes between the green and magenta cells indicated by the yellow arrows, and reaches the rear of the cluster within ∼10 min. The gold cell, moving along the blue dotted arrow, has similar behaviour and moves between the two green cells indicated by the white arrows. Supplementary Movie 6 illustrates this example of the neighbour exchange. At time point=23 in this video, the shape of the gold cell nucleus is elongated probably because it was squeezed by the two green cells. During the neighbour exchange, the topological arrangement of the other cells remains largely unchanged. We can glean some information about the relative position of a cell by observing the interface with its neighbours. However, this is insufficient to fully describe neighbour exchange. To obtain a complete picture of moving cluster, we must identify neighbour exchange based on global topological change. We designed a temporal topology change metric in Methods and results are shown in Fig. 4b–d. The *x* axis shows when a neighbour exchange happens and the *y* axis shows how long it will take. Results for two representative rotating and running movies without any neighbour exchange are shown in Fig. 4b,c (Corresponding reconstructed movies are shown in respectively). A movie with neighbour exchange is shown in Fig. 4d (Reconstructed movie shown in Supplementary Movie 6). The local maxima in Fig. 4d, the hot spots indicated by the annotated arrows, show that this movie has four events of neighbour exchange. The local maxima highlighted by the red arrow in Fig. 4d suggests that this neighbour exchange takes about 6–7 time points at around time-point=20 (The gold cell nuclei in Fig. 4a). Overall, the relative positions of the border-polar cells are stable during both running and rotating, but it is possible for border cells to slide along the surface of polar cells and exchange their position with other border cells.

### Cluster external protrusion and internal cell extension

During cluster migration, border cells may generate elongated protrusions extending out along the norm direction from the main body of cluster (Fig. 2e meshed surface highlight by the red arrows). The definitions of different types of cell protrusions are summarized in ref. 4. However, there are not yet clear mathematical or morphological criteria to differentiate them. We describe a morphology operation in Methods to detect and measure these functional structures. Our computationally defined protrusions may not exactly correspond to cell biology defined F-actin filled protrusions. We designate them as cluster external protrusions but without further classification into different sub-categories. The cluster external protrusion is a dynamic structure at the whole cluster level and it can take up to 50–70% volume of a border cell, potentially including the volume of the nucleus.

On the basis of our segmentation and tracking, it is possible to accurately describe the deformation of an individual border cell over time. The dynamics of cell surface deformation was previously inaccessible but is a key aspect to understand single cell behaviours in a cluster. To quantify this deformation over time, we assume the sampling interval of image acquisition is small enough and thus provide a linear mapping solution in the Computational 3D Morphological Dynamics section of Methods. Besides the massive cluster extensions, border cells display more individual behaviours and can generate cell extensions along the tangent direction of the cluster. We summarize these two types of protrusions as follows. A cluster external protrusion is an elongated protrusion detected using morphology operation along the norm direction of the cluster. It often makes contacts with two or more nurse cells but not other border cells. Typically, these protrusions are visible at the cluster level, squeezing in between the interface of nurse cells. A cluster internal cell extension is an individual cell extension, detected by computational deformation analysis. Individual cells produce extensions along the tangent direction of the cluster. These extensions normally occur at the interface between other border cells and nurse cells. In this case, a border cell is observed ‘reaching around' another cell. Such behaviour plays an important role in the neighbour exchange events as illustrated by Supplementary Movie 9.

### Cluster external protrusion and running mode

**Figure 5:**
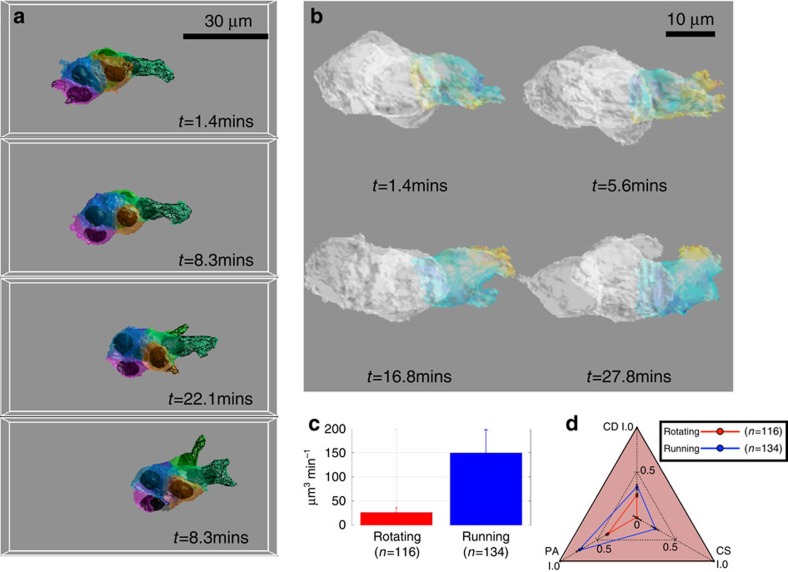
Cluster external protrusions are associated with cluster running. (**a**) Cluster external protrusion detection and measurement at four different time points. The meshed surface indicates the cluster external protrusion and the color indicates which cell generates the cluster external protrusion. (**b**) The morphological dynamics of a leading cell of a running cluster (Supplementary Movie 11). Hotter color indicates bigger deformation. (**c**) Quantification of cluster external protrusion in rotating and running modes. During rotating mode, the cluster generates ∼25 μm^3^ min^−1^ protrusions, while during running mode it can generate much larger protrusions of 150 μm^3^ min^−1^. Error bar is the Standard Error of the mean. (**d**) Cluster external protrusion parameters differ during rotating and running modes. PA: alignment of each cluster external protrusion with cluster migrating direction, protrusion directions are first normalized, and the magnitude of the unit vector's dot product with the cluster direction (also normalized) is taken as PA. In running phase, the protrusions are more highly correlated with the cluster direction, *i.e.* the cluster moves along the cluster external protrusion direction. CD: the normalized protrusion direction vectors are added as a combined vector and its magnitude is correlated with that of the cluster speed as CD. This parameter indicates if the combined protrusion direction associates with the cluster speed magnitude. Again, CD is higher for running than rotating. CS: the correlation between protrusion mass and cluster speed, indicating whether the size of a protrusion is associated with cluster speed.

Cluster external protrusions are illustrated by the meshed surface in Fig. 5a and Supplementary Movie 9. The morphology dynamics of the cluster external protrusion is shown in Fig. 5b and Supplementary Movie 10. Using our method, we can identify which cell produced the protrusion and quantify the protrusion volume (μm^3^) and its direction. The generation of cluster external protrusions is very different during running and rotating modes in Fig. 5c. The average cluster external protrusion rate is ∼25 μm^3^ min^−1^ for rotating mode, while the cluster can generate massive cluster external protrusion at 150 μm^3^ min^−1^ during running mode.

In what follows we provide analysis on the different roles of cluster external protrusion in running and rotating modes as presented in Fig. 5d. The first parameter is the alignment of each cluster external protrusion direction and the cluster migrating direction. The protrusion directions are first normalized, that is, only considering the direction as a unit vector without magnitude information. Each vector conducts a dot product with the cluster direction (also normalized). We take the magnitude of the dot product as protrusion alignment (PA) with cluster direction in Fig. 5d. If the protrusion direction perfectly aligned with cluster migration direction, PA=1. When opposite, PA=−1. During rotating mode, the cluster external protrusion direction can show correlation with the cluster movement direction, that is, the PA is <0.4, the correlation is much higher during running mode, PA is >0.7. The normalized protrusion direction vectors are added as a combined vector. Then we applied correlation analysis between this vector's magnitude and the cluster speed magnitude as the impact to cluster direction (CD) in Fig. 5d, that is, combined directions versus cluster speed magnitude. Then we conducted correlation analysis between the two variables (see Computational 3D morphological dynamics in Methods section). This parameter indicates whether the combined protrusion direction correlates with the cluster speed magnitude. CD is also higher during running mode in Fig. 5d. External protrusions are therefore less related to forward movement during rotating than running mode. Finally, we measure the correlation between the protrusion mass and the cluster speed magnitude, as the impact of protrusion size to cluster speed (CS). In rotating, the cluster external protrusion has almost no correlation to cluster movement, while it much more highly correlated during running mode as shown by CS in Fig. 5d.

### Cluster internal cell extensions and rotating mode

**Figure 6:**
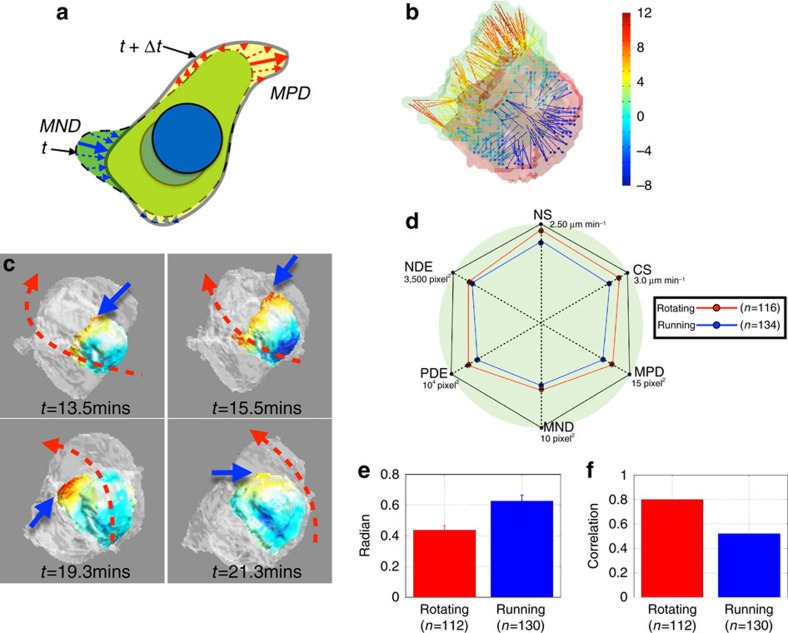
Cluster internal cell extensions are associated with cluster rotation and cells are more active during the rotating modes. (**a**) Illustration of morphodynamics parameter calculations: the Maximum Positive Deformation (MPD), Maximum Negative Deformation (MND), Positive Deformation Energy (PDE) and Negative Deformation Energy (NDE). The solid red arrow is the MPD and the blue solid arrow is the MND. The PDE is the summation of all red arrows and the NDE is the summation of all blue arrows. (**b**) Morphodynamics of a border cell in 3D. Hot color indicates positive deformation and cold color indicates negative deformation. (**c**) Cluster internal cell extensions are associated with cluster rotation. Blue bold arrows indicate the cluster internal cell extensions of a border cell and the red dotted arrows indicate the rotating direction of the cluster. (**d**) Six measured parameters, including Nucleus Speed (NS), Cell Speed (CS) and those defined in **a** reveal that the cells are more active during the rotating mode than the running mode. Nucleus and cell speed are higher, and the cell front edges are more active, generating larger cluster internal cell extension during the rotation, while the retraction behaviour remains similar, indicated by the MND and NDE. (**e**) The angle difference between the cluster rotating vector (red arrow in Supplementary Fig. 12) and protrusion rotating vector (magnet arrow in Supplementary Fig. 12). The difference between these two vectors is often <30 degrees in rotating mode but much greater in running mode. Error bar represents s.e.m. (**f**) The correlation between the rotating speed and the positive protrusion amount. The correlation is about 0.80, compares to only 0.51 in running mode. This indicates cluster rotating behaviour is highly associated with the well-coordinated cluster internal protrusions.

The deformation of individual cells within the cluster may provide us with additional insights into cluster coordination. In the Computational 3D Morphological Dynamics section of the Methods, we provided a linear mapping solution to estimate the deformation of a given cell between two consecutive time points. A 2D illustration of our linear mapping is given in Fig. 6a. The actual 3D deformation of a border cell is illustrated by color-coded vectors in Fig. 6b. Hot color indicates positive deformation and cold color indicates negative deformation. During the running mode, the leading cell front edge is also very active, as shown in Supplementary Movie 11. Similarly, in the rotating mode, the cluster internal protrusion is detected by the positive deformation as indicated by the blue arrows in Fig. 6c. During rotating mode, the cells generate cell extensions between the interface of the nurse cell and border cells as seen in Supplementary Movie 12. The morphodynamics and the deformation measurement of a border cell within a rotating cluster are shown in Fig. 6c. The cluster internal cell extensions are associated with the cluster rotating direction indicated by the red dotted arrows. We hypothesize that uncoordinated cluster internal extensions, as illustrated in Supplementary Movie 7, would also provide a mechanism for individual and uncoordinated intra-cluster motility, such as the described neighbour exchange.

Supplementary Fig. 11a,b show the positive deformation of all cells in the running and rotating cluster respectively. The positive deformations are shown in color-coded solid line and the negative deformations are in the dashed color line. The deformation of the front (positive deformation) is much bigger than rear edge (negative deformation) of a given cell in both running and rotating modes. We also observed that the dynamics calculation, positive deformation energy (PDE), as defined in Methods, and shown in Supplementary Fig. 11, has a periodic behaviour with a frequency of ∼3–4 min. However, our current temporal resolution does not allow a conclusive statement. Better temporal and spatial resolutions are required for further investigation.

Consistent with previous reports we show that the cluster speed is lower in rotating mode than running (Supplementary Fig. 7). However, it is not clear if the individual cells move more slowly during the rotating mode. Our computational analysis reveals, counter-intuitively, that the cells are slightly more active in the rotating mode than the running mode based on 6 parameters defined in Methods section (Fig. 6d).

The association between cluster internal protrusions and cluster rotation is also validated. The rotating movement of the cluster takes into consideration all cell movements (represented by the nuclei center displacement vector) indicated by the blue arrow in Supplementary Fig. 12. The direction of a given cell to the cluster center is indicated by the red dashed line. The cross product of these two vectors gives a perpendicular vector and the combination of all those perpendicular vectors of all cells produces a combined cluster rotating vector indicated by the red arrow in Supplementary Fig. 12. Similarly, the cell positive deformation represents the cluster internal protrusion, illustrated by the green arrows in Supplementary Fig. 12. Applying the same computation to those vectors, we can get a combined protrusion rotating vector, represented by the magnet arrow in Supplementary Fig. 12. First, we checked the angle between combined cluster rotating vector and the combined protrusion rotating vector. The results of running and rotating mode are shown in Fig. 6e. We can see that the angle between these two vectors is <30 degrees in rotating mode but much greater in running. We also validate that the rotating speed, represented by the magnitude of combined cluster rotating vector, is correlated with the protrusion amount, represented by the magnitude of combined protrusion rotating vector, in Fig. 6f. The correlation between these two parameters is about 0.80, compares to only 0.51 in running. Thus the cluster rotating behaviour is highly associated with the well-coordinated cluster internal protrusion. At times the cluster internal protrusion is not coordinated, as shown in Supplementary Movie 9 and such behaviour is associated with neighbour exchange in the cluster.

## Discussion

The *Drosophila* border cell cluster is an excellent *in vivo* system to study collective cell migration. Quantitative analysis of the collectively migrating cells in full 3D is necessary to better understand the complex behaviour of individual cell and the cluster as a whole, but an appropriate computational solution has been lacking. Here we present a computational method including image pre-processing, segmentation, tracking and quantitative information extraction and data interoperation.

Although our computational solution is optimized, segmentation is somewhat limited by the imaging techniques. Although we may image the cluster with reasonable spacial and temporal resolution, we are still limited by current microscopy techniques. Typical cell membrane width is about 0.01 μm and such fine structure is not resolvable with the current imaging setup. To balance the spatial and temporal resolution, while reducing potential photo-toxicity, we can only achieve limited spatial resolution. A typical border cell has a volume of ∼1,000–1,500 μm^3^ (∼20,000–30,000 voxels). This gives us an estimated 3D object with a radius of only 17–20 voxels. Although we enhance the weak membrane signal, segmentation errors may still happen. As the watershed is a method using the gradient of the signal. Both noise and other factors may affect the real gradient of the signal. For example, when two membranes of the same cell are very close, the nearby signal may affect the reading. Within the above imaging our method resolves nuclei, cell surfaces and their morphology as shown in Fig. 1.

The tracking of moving nuclei and cells is still a challenging step too, especially when cell speed is highly variable. Although the nuclei and cells can have significantly higher speed at some time points, the proposed tracking solution is able to provide us a metric which can be optimized using association algorithms and promises a global minimum. Based on our results, conventional parameters, such as volume and speed, can be extracted in the real 3D context on a cell-by-cell basis (Fig. 2). We observe that cell speed is generally higher than nucleus speed (Supplementary Fig. 4). The cell membrane is more active than the nucleus, which generally remains in a round or spherical shape. The slower speed of the nuclei is caused by the fact that the cell membrane is deforming, squeezing and moving forward, and hence displacing the cell's center of mass while the nucleus does not follow immediately. We observed two possible scenarios (Fig. 2d). First, the membrane is deforming and moving, while the nucleus does not move much. This will cause a difference between cell and nuclei speed. Second, both the membrane and the nucleus move together with a higher speed.

Previous qualitative observations of highly coordinated and chaotic movement of the cluster have been reported. The coordinated movement can be deconvolved into two distinct modes, running and rotating. These can be quantitatively described and classified using an experimental boundary. Discrete switching between these two modes occurs infrequently. The running and rotating modes in this *in vivo* study are very similar to a previous *in vitro* work^20^ and similar collective behaviour has also been reported in schools of fish^21^. Generally, the motion of the cluster can be described as changing combinations. The running mode tends to dominate as the cluster initiates migration, whereas the rotating mode dominates as the cluster nears its destination^2^,^8^. In addition, the border cell cluster has some chaotic statuses between the rotating and the running modes, which remain difficult to distinguish from coordinated movement (Supplementary Fig. 8). Indeed, our quantitative analysis suggests a continuum of migration states between simple running and rotating classifications as in Fig. 3b,c. Our work also suggests that while simple parameters such as cluster speed may be useful to measure, additional insight can be gained by looking at the frequency of neighbour exchange using Angular Momentum and Group Polarity.

We demonstrate the possibilities enabled by our image analysis solution, and can make a few novel insights into the border cell migration. First of all, we show that polar cells rotate with the whole cluster during the rotating mode, while individual border cells are able to slide over the polar cell surface and exchange position with their neighbours (Fig. 4). Second, defining the surfaces of the individual nuclei/cells is crucial to understand the dynamics of cell-cell coupling and the degree of the coordination. Even without neighbour exchange, border cells can significantly change the contacting surface with other border cells during migration (Fig. 2f,g). Third, we are able to measure cluster external protrusions and quantify their correlation with cluster motion during running and rotating modes (Fig. 5). Finally, we identify another class of extensions, cluster internal cell extensions, which cannot easily be observed or quantified in 2D analysis at the whole cluster level (Fig. 6c). These extensions ‘reach around' neighbouring border cells, and are associated with rotation of the cluster and neighbour exchange. Our 3D analysis at single cell resolution also allows us to show that the cells do not lose their mobility during the rotating mode. Study of cell membrane dynamics and the associated force is the key to understanding collective cell migration. Some solutions have been proposed for the computation of cell membrane deformation in 2D^22^,^23^. Based on our assumption that the sampling interval is small enough, we applied the linear mapping solution between two consecutive time points to estimate the deformation of a given cell in 3D. On the basis of our 3D morphological dynamics data, border cells are slightly more active in rotating than running. We hypothesize that during the rotating mode, the cluster may encounter less friction when rotating within the space it occupies than when running, in which the cluster must overcome the frictions at the interface between nurse cells to occupy new space. It is also possible that the rotating mode represents a highly dynamic stage of signal sensing between more progressive running modes. Further work will be required to test these hypotheses, for example by measuring tension in the different modes or using signaling pathway reporters to assess differences in signaling states. The mechanism by which the cluster transitions between the running and rotating modes remains unclear but the approaches described here provide a basis for its further investigation.

Our dedicated solution, the CCMToolkit, provides a valuable new set of tools to automate the study of collective cell migration in 4D. It is freely available at (https://sites.google.com/site/ccmtoolkit/) with relevant technical documents, developer's API and testing data set. While our initial work has focused on wild type clusters, using these extra parameters, it will be possible to characterize different mutations and RNAi or overexpression phenotypes in more detail. For example, mutations which reduce border cell–border cell adhesion may display larger changes in neighbour interfaces, and may display higher rates of neighbour exchange. Taking an automated, computational approach will allow more objective interpretation of mutant behaviours and allow us to cluster mutant types based on behavioural similarities. Application of CCMToolKit to wild type and mutant border cell clusters has the potential to offer novel insights into the mechanisms of collective cell migration. In general, this solution is applicable to other cell migration and tracking problems subject to optimization of imaging protocols.

## Methods

### Fly genetics and imaging approach

UASt-PH(PLCdelta)-GFP, UASt-histone-RFP, Upd-Gal4 and Slbo-Gal4 are described in Flybase [Flybase.org]. UMAT-Lyn-tdTomato was constructed by inserting the membrane-targeted Lyn-tdTomato fusion (gift from Darren Gilmour) into pUMAT (containing the maternal alpha tubulin promoter, gift from Daniel St. Johnston) and transgenic flies were made by Bestgene. Slbo-Gal4 drives UAS transgene expression in outer migratory border cells, and Upd-Gal4 in polar cells. Together they allow expression of the nuclear marker (histone-RFP) and a cell membrane marker (PH-GFP) in all cluster cells. UMAT-Lyn-tdTomato marks nurse cell and oocyte membranes.

For two-marker videos: UpdGal4/+;;UAS-PH-GFP,slboGal4/UAS-his-RFP. For three-marker videos: UpdGal4/+;UMAT-lyn-tdTom/+;UAS-PH-GFP,slboGal4/UAS-his-RFP. For videos with single (outer) border cells marked: hsFLP/+;;2x(tubp-GAL80), FRT80,slboGal4/UAS-PH-GFP,UAS-his-RFP,FRT80. For videos of marked polar cells: UpdGal4/+;; UAS-PH-GFP,UAS-his-RFP/+

Dissection and live imaging of egg chambers was performed in imaging medium (Schneider's medium supplemented with 2.5% FCS, 5 μg ml^−l^ insulin, 2 mg ml^−l^ trehalose, 5 μM methoprene, 1 μg ml^−l^ 20-hydroxyecdysone, 50 ng ml^−1^ adenosine deaminase and 9 μM FM 4–64) as described in ref. 24. Imaging was performed using am inverted confocal microscope (Leica SP5) For three-marker videos, four channels were acquired simultaneously: GFP (488 nM laser, collect emission 505–550 nm), Red 1 (561 nm laser, collect emission 570–620 nm), Red 2 (561 nm Laser, collect emission 620–790 nm) and transmission image (DIC). tdTomato and dsRed signals were unmixed using a linear unmixing approach. This was implemented using ImageJ and the Spectral Unmixing plugin written by Joachim Walther. The egg chamber drifting was corrected using the method in ref. 25. For two-marker videos, red 1 and red 2 channels were combined for RFP signal. The movies were recorded by using an inverted confocal microscope (SP5; Leica) with a 63 × , 1.2 NA Plan Apochromat water immersion objective with 2.5 zoom factor. The resolution is 296 × 150. Sampling intervals were 1–1.5 min and imaging continued until the border cells touch the edge of the imaging volume or a maximum of 2 h.

### Image reconstruction and pre-processing

Confocal microscopy is able to acquire 3D time-lapse videos, however the image stacks may have some quality issues. The challenges include the following aspects: (a). Acquired image stacks are of low signal-noise-ratio. This is because moderate laser power is selected to minimize the photo-bleaching; (b). Despite moderate laser power, photo-bleaching still occurs, especially for the nuclei channel (Supplementary Fig. 1); (c). Anisotropic voxel size of different directions caused by: (i) The digital motor step in the *z* axis is generally larger than the *x–y* resolution; (ii) point spread function (PSF) has less optical resolution in the *z* axis.

Proper pre-processing is needed to address those quality issues. First of all, voxel size of acquired image stacks is different in different directions and we need to reconstruct them. The voxels size is normalized using linear interpolation and the scales of voxels are then isotropic post-reconstructed, that is, each voxel is 0.33 × 0.33 × 0.33 μm. This step compensates for the bigger motor step in z direction, but cannot solve the issue of optical PSF. Based on the assumption that the acquired image contains a constant amount of florescence, the photo bleaching of all channels are compensated. The corrections of fluorescent energy for different channels are shown in Supplementary Fig. 1. This step potentially also amplified the noise at the latter stage of a movie. To reduce the noise, image stacks are smoothed by Gaussian kernel. Non-uniform background removal is also applied.

In our study, there are two types of cells within the migrating cluster: (i) A pair of polar cells that are in the center of the cluster; (ii) A fixed number of border cells surrounding the polar cells.

The only manual work in our analysis is to annotate the center of these two types of cells in the first image stack. For this we designed an annotation package named ‘CCM Annotator' for this step. The remaining steps are fully automatic, including nuclei segmentation based on GMM, nuclei/cell tracking using a global cost function and cell segmentation based membrane signal.

### Nuclei segmentation based on GMM

While previous efforts have been made on cell/nuclei segmentation and tracking it remains a challenging task. Existing segmentation approaches for nuclei segmentation, include simple thresholding^26^, watershed approach^27^,^28^, iterative voting methods^29^, level set approach based on gradient flow^30^ and flexible contour model^31^. The watershed approach has been widely applied for cell segmentation. Different solutions have been proposed to overcome over-segmentation, for example, rule-based merging^32^ and marker-controlled Voronoi diagram^33^, a level set formulation for Watershed segmentation^34^, preserving topology by simple point concept^35^. Some recent approaches are reported, such as multiple level set function for individual cells^36^ and topological dependence^37^,^38^,^39^. Tracking is particularly challenging. Two basic point-based tracking techniques are centroid tracker^40^ and correlation tracker^41^,^42^. Other tracking approaches include snake model with gradient vector flow^43^, snake model with shape and size constraint^44^,^45^, active contour approach for both segmentation and tracking^46^, K-mean clustering and matching^47^, and Gaussian Mixture Model^48^,^49^. Other relevant works on 3D time-lapse analysis include adaptive recursive analysis based on iterative thresholding^50^, blob detection based on Laplacian of Gaussian^51^, and a constrained active contours approach^52^. As we mentioned, application of those algorithms to the border cell system is not trivial and we are in need of a complete and automatic solution.

The *a priori* knowledge on the fixed cell number is a major challenge and we do not tolerate any segmentation or tracking error in a movie of typical ∼100 time points. Furthermore, we need to identify the location and boundaries of the nuclei and cells. Since the nuclei are closely packed with each other, a Laplacian kernel is applied to enhance the boundaries between them.

From the annotation at the first time point, we know the number of nuclei and their identities, that is, polar or border cell. We denote a fixed number of nuclei using *ω*_1_, *ω*_1_,…*ω*_*c*_, where *c* is the total number of nuclei. A threshold and some morphological operations are applied to obtain the foreground of the nuclei, for example, a mask covering all nuclei. During this process, we conserved the total mass of all nuclei.

For each voxel belonging to the foreground, we may form a vector, which is given by:


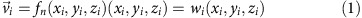


Where 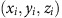 are the coordinates of the given voxel and 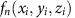 is the brightness at the voxel in the nuclei channel, denoted by 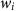. 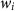 is just a weighting parameter for this coordinate. In total we have *n* such samples giving 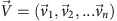, where *n* is the total number of voxels in the mask. The segmentation of nuclei now becomes a task to assign a unique label to each sample from 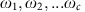.

We assume that the samples are drawn independently and identically from the distribution of 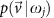, which has a known parametric form that is uniquely determined by a parameter vector 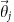. Explicitly, we write 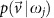 as 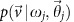. Here independent means the samples from different state *ω*_*j*_ give no information about 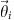 if 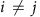. Here our assumption is that the parameters for different states are functionally independent. In our study, we have 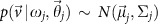 and 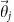 includes both 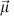 and 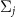. Thus our observation 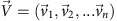 is drawn from a Gaussian Mixture Model (GMM), given by:


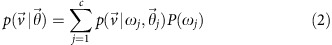



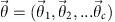
 is an unknown stationary parameter vector that we want to estimate from observations. In principle, if 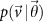 is identifiable, 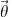 can be recovered from empirical observations. Here identifiable indicates there exist a 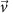 such that 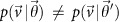 if 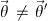. The mixture of norm density is usually identifiable. The likelihood of observed samples is a joint density:


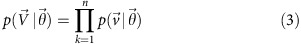


The maximum likelihood (ML) estimation of 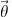 is the value of 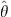 that maximizes 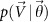 in Equation (3). As we already know the number of cells, we only need to fit our data with the given model.

As we assumed 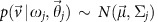, thus 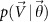 is differentiable with respect to 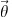. We can actually derive the analytical necessary condition for 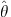 since we know the gradient respect to 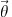 must vanish at 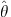. However, when both 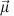 and 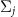 are unknown in our study, the ML solution is singular. Although the number of the nuclei is fixed, we can let the variance approach zero thus make the likelihood arbitrarily large. Even so, a useful solution can still be obtained when placing meaningful constraints on the covariance matrix to ensure positive definiteness of the obtained covariance matrix. Since we are sure that the values at the diagonal entries of each covariance matrix should not be smaller than some value, a tiny positive real can be added to the diagonal entries as a regularizer to enforce this prior constraint. Or we can restrict ourselves to work with only diagonal covariance matrices. In practice, the classical expectation-maximization (EM) algorithm is considered to obtain the local maximal solution of the likelihood function in Equation (3). Once the ML solution for 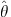 is obtained, in our context we have ready each of the components 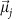 as the weighted geometric center of each nuclei, together with the corresponding shapes described by 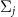.

### Nuclei/cell tracking with global cost function optimization

The tracking of nuclei is a problem of association or matching at different time point. It can be solved using Markov Chain or traditional point match algorithms. In this paper, we propose an appearance based tracking model using a global cost function optimization.

Let's say the nuclei segments at time *t* and *t*+1 are denoted by 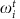 and 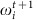 respectively, where 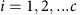. We can define a matrix of tracking confidence and each element in this matrix is given by:


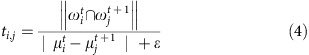



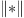
 in the numerator means the norm of a set of voxels, which give us the overlapped volume of two segments at two different time points. || in the denominator means the Euclidean distance between the nuclei centers at two time points. Intuitively, Equation (4) tells us if the distance between two segments is smaller, they are more likely associated and it also favours the segments, which have greater overlapping volume. *ɛ* is a regulator to increase the stability of the matrix.

In building such a matrix of tracking confidence, the nuclei tracking becomes an assignment problem. Given two sets 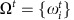 and 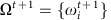 of equal size, together with the tracking confidence 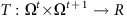, which elements are defined in Equation (4). We hope to find a bijection (also known as one-to-one matching.)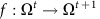 such that cost function 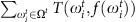 is maximized, which gives us:


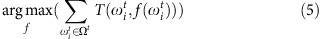


Equation (5) means that of all possible matches, we hope to find a perfect match, which maximizes the summation of our tracking confidence at 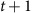. This problem can be efficiently solved using combinatorial optimization algorithms, such as the classical Hungarian algorithm. This guarantees an optimal solution for the cluster of cells, instead of just using the local information for nuclei tracking. As we can see in Supplementary Movie 1, even when some cells move very fast at certain times, we can still track them successfully using our approach.

### Marker controlled cell segmentation using membrane signal

We know that the cluster and/or individual cells do not suddenly gain or lose mass during the migration process. Constraining the volume of each individual cell is theoretically feasible, but given the limitations of image acquisition such a solution may not bring us more benefit and is much more computationally expensive. In our work, we used a greedy algorithm to determine an optimal thresholding value which provides a mask for the cluster with a volume determined by the first time point.

The membrane signal is relatively weak and lacking in specificity so we used a rotational kernel to enhance the membrane signal based on the infinity norm. Since the nuclei are already segmented and tracked, we can then use them as seeds to apply a seeds-controlled watershed algorithm to segment the cells based on the enhanced membrane signal.

### The coordination of nuclei/cell movement

After nuclei tracking, we can quantify nuclei movement. Let's say nucleus 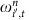 at time point *t* matched to 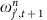 at time point *t*+1. Their geometric centers are represented by 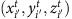 and 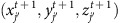, then we may calculate a vector to represent the movement of each nucleus:


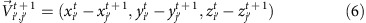


The displacement, that is, magnitude, between two time points:


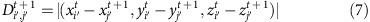


Where |*| means norm. Note that 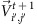 is a vector and its magnitude is given by


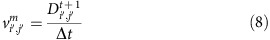


The direction of 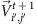 can be represented by a normalized vector:


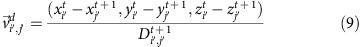


Note that 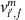 is scalar, but 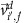 is a vector with norm equal to 1. At each time point, we will have a number of tracking identities. The concept of velocity is a vector which is represented by its magnitude and direction. For simplicity, let's use 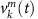 and 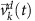, where 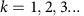, to represent its magnitude and direction respectively.

We visualized the nuclei velocity direction with the original image stacks. We observed clearly two different migration statuses, called running mode and rotating mode, illustrated in Fig. 3b,c. Furthermore, we calculated two features, named Group Polarization and Angular Momentum, for a quantitative understanding of these two different migrating statuses.


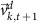
 is a vector only representing the direction of each nucleus' movement and contains no magnitude information. We interrogate the question of whether the nuclei move in the same direction at each time point or are moving in different directions. To answer this question, we may calculate the summation of 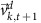 at each time point and denoted it as Group Polarization:


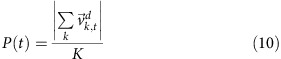


Where K is total number of 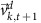 and 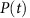 is a scalar between [0 1]. If the cluster is in the running phase, then 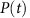 is close to 1.0 as shown in Fig. 3a. The value of 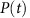 will be much lower when the cluster is under rotation phase. The calculation of 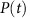 is represented by the red curve in Fig. 3a.

To identify the rotation movement of the cluster, we further define the angular momentum of the cluster. For each nucleus, its center position is denoted as 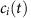. The cluster center is then given by


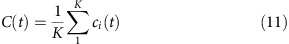


We obtain the vector of the nuclei to the cluster center as:


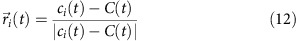


Note that 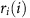 is a normalized vector. The angular momentum is given by:


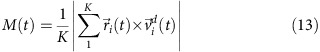


Again *K* is total number of 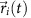 and 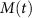 is a scalar between [0 1]. If the cluster is in the rotation phase, 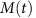 will be close to 1.0 as shown in Fig. 3a. The value of 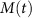 will be much lower when the cluster is under running phase. The calculation of 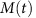 is represented by the blue curve in Fig. 3a.

### Neighbour exchange of border cells during migration

Another interesting behaviour we aimed to quantify is neighbour exchange. An example of which is shown in Fig. 4a. The nucleus/cell center position, denoted by 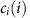, can provide such information. We first calculate the inter-cell distance matrix at a given time *t* as 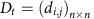. Here 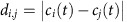. Let 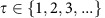 be the temporal stepsize, and define the temporal difference of step-size 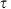 at time point as:


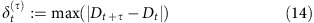


With 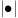 denoting element-wise absolute values and max(.) being the max operation over the matrix element.

Empirically the measurement 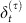 are plotting over time t and the temporal scale spaces 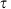. Denote the cut-off thresholding 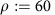. Clearly most of the neighbourhood exchange cases have very high values (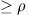), while most of the rotation and sliding cases have relatively low values (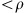). This suggests 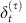 is a good parameter to detect the neighbourhood exchange during collective cell migration. It is worth noting that the threshold 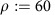 is about the twice the average nucleus diameter (30 voxels) in our context. Meanwhile, the temporal scale space offers new insights into how the neighbourhood exchanges could be organized over time.

### Cluster external protrusion detection and analysis

The protrusions formed by the border cells play an important role in the migratory process. There are some specific biomarkers for different protrusions but these are difficult to implement in the *in vivo* experiments. Here we use a morphological operation to detect cell extensions. Typically, a cluster external protrusion is a structure extending along the normal direction of the cluster surface and its thickness is smaller than the diameter of the cluster. Though cluster external protrusions are cluster level structures, they are still generated by individual border cells. We calculated the averaged volume of the border cell and use this volume to fit it as a sphere. The radius of this sphere is then used for a morphological operation, called opening, which is given as follows. If the binary image of the cluster is given by *B*, and the ball structure element is given by *S*, then the detected cluster external protrusion is:


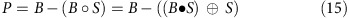


Where 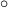 is the opening operation, which is erosion, that is, ‘·', followed by dilation ‘^⊕^' of the given element ball structure of radius *r*. Some parameters of the detected cluster external protrusions are measured. Cluster external protrusion size is calculated by 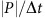, where |*| is taking the norm of the protrusion, that is, its volume and 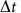 is the sampling interval between the two image stack. The normalization according to the sampling interval is necessary, as if we see a bigger protrusion size, we are not sure that if it is caused by more active front cell behaviour or it has longer time to generate bigger protrusion. The cluster external protrusion's direction is measured according to difference between the cluster mass center and the protrusion mass center. During the tracking, the speed of cluster mass center is calculated as follows. After the opening operation, that is, 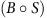, we actually take away the elongated protrusions, and the rest is a more spheroid ball, we called cluster body. Let **m**(*t*)=(*x,y,z*) is the position of cluster body mass center, **m**(*t*+1)=(*x′,y′,z′)* is for t+1. The **d**(*t*) is the displacement between **m**(*t*) and **m**(*t*+1). Then cluster speed is given by **v**(t)=**d**(t)/*Δt*. The direction of this vector defines the cluster direction. The detected cluster external protrusions of a given movie is shown in Supplementary Movie 10.

The correlation is a standard parameter to measure how strongly two variables are correlated. Let's say the two variables are *X*(*t*), that is, combined directions and *Y*(*t*), i.e. cluster speed magnitude. The correlation matrix is calculated in this way: Σ=(*E*(*X*−*μ*_*x*_)(*Y*−*μ*_*y*_))/(*σ*_*x*_*σ*_*y*_). Where *μ*_*x*_ and *μ*_*y*_ is the mean of *X* and *Y*; *σ*_*x*_ and *σ*_*y*_ is the standard deviation of *X* and *Y*. Note that Σ is a 2 × 2 symmetric matrix. The diagonal elements are the correlation coefficient between *X* and *Y*. If this correlation is equal to 1, it means the two variables are positive linearly correlated; if it is zero, it means the two variables have no relationship to each other. Supplementary Movies 11–12 illustrate the deformation of the border cells in running and rotating modes.

### Computational 3D morphological dynamics

In our experiments, the high temporal resolution of the raw data as well as our accurate segmentation provided us with a basis to look at the dynamics of each migrating cell surface over time in 3D, that is the morphodynamics. The movement of the cell is a continuous process and the dynamics of its surface contains a lot of information. Some methods are developed to explore the cell membrane deformation in 2D are reported^22^,^23^. In our 3D study, it is to build an association of two cell surfaces between two consecutive time points. Once we achieve this, we can then estimate the deformation along the cell surface at two different time points. The retraction and forward extension of the cell can thus be quantitatively described. For a migrating and deforming cell, its surface can be described as a function, that is, 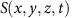. In Fig. 6b, we demonstrate our 3D computation in 2D. As we have excellent temporal resolution, we assume that any deformation along the norm direction of a cell surface is small enough that we can treat it as linear. The deformation between any two time points is given by:


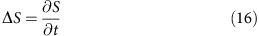


On the basis of our linear assumption, Equation (16) is actually to find a linear minimum distance mapping for the points on the surfaces at two different time points. The arrows in Fig. 6b, show the matching points. We also need to define whether the deformation is positive (extending) or negative (retracting). Positive deformation means a surface moving into a space which was not previously occupied by the cell, shown by the red arrows in Fig. 6b; while negative deformation means a surface retracting into a space which was previously internal to the cell, shown as the blue arrow in Fig. 6b. The actual deformation calculation of a given cell is shown in Fig. 6c. In our work, we take the positive deformation of the cell surface as the cluster internal protrusion.

To quantify the cell mobility, we defined the following 6 parameters. Nuclei speed: represented as ‘NS' axis Fig. 6d; cell speed: represented as ‘CS' axis in Fig. 6d; maximum positive deformation: The maximum deformation vector size in the positive deformation vectors, shown as the solid red arrow in Fig. 6a represented as ‘MPD' axis in Fig. 6d; maximum negative deformation: The maximum deformation vector size in the negative deformation vectors, shown as the solid blue arrow in Fig. 6a represented as ‘MND' axis in Fig. 6d; positive deformation energy: The integration of all positive deformation, that is, the summation of all red arrows as shown in Fig. 6a, represented as ‘PDE' axis in Fig. 6d; negative deformation energy: The integration of all negative deformation, that is, the summation of all blue arrows as shown in Fig. 6a, represented as ‘NDE' axis in Fig. 6d.

### Data availability

The data sets generated during and/or analysed during this study are available in the website of CCMToolKit (https://sites.google.com/site/ccmtoolkit/). Both source code and a few example movies are provided. Other further information and details are available from the corresponding author on reasonable request.

## Additional information

**How to cite this article:** Cliffe, A *et al*. Quantitative 3D analysis of complex single border cell behaviors in coordinated collective cell migration. *Nat. Commun.* **8**, 14905 doi: 10.1038/ncomms14905 (2017).

**Publisher's note:** Springer Nature remains neutral with regard to jurisdictional claims in published maps and institutional affiliations.

## Supplementary Material

Supplementary Information Supplementary Figures

Supplementary Movie 1a Reconstructed 4-D movie with tracked nuclei and cell.

Supplementary Movie 1b Reconstructed 4-D movie with tracked nuclei and cell.

Supplementary Movie 2 Visualization of segmentation results of a given 3-D image stack at different z-slices.

Supplementary Movie 3 The migrating cluster of running mode. Yellow line indicates polar axis, a straight line passing through the mass centre of the two polar cells.

Supplementary Movie 4 The migrating cluster of rotating mode. Yellow line indicates polar axis, a straight line passing through the mass centre of the two polar cells.

Supplementary Movie 5 Comparison of polar axis rotation between running and rotating mode.

Supplementary Movie 6 Reconstructed movie with neighbor exchange. (Temporal Topology Change Metric as shown in Fig. 6d).

Supplementary Movie 7 Reconstructed movie without neighbor exchange (Temporal Topology Change Metric as shown in Fig. 6b).

Supplementary Movie 8 Reconstructed movie without neighbor exchange (Temporal Topology Change Metric as shown in Fig. 6c.).

Supplementary Movie 9 The detected cluster external protrusion.

Supplementary Movie 10 Deformation of a given border cell within the moving cluster undertaking the neighbor exchange.

Supplementary Movie 11 Deformation of a given leading border cell within the running cluster.

Supplementary Movie 12 Deformation of a given border cell within the rotating cluster at different time points.
