# Supplementary material for: Quantitative 3D analysis of complex single border cell behaviors in coordinated collective cell migration
Source: Nat Commun. 2017 Apr 4;8:14905. doi: 10.1038/ncomms14905 (PMC5382290; doi:10.1038/ncomms14905)
Supplement: Supplementary Information — Supplementary Figures [file ncomms14905-s1.pdf]

Supplementary Fig. 1

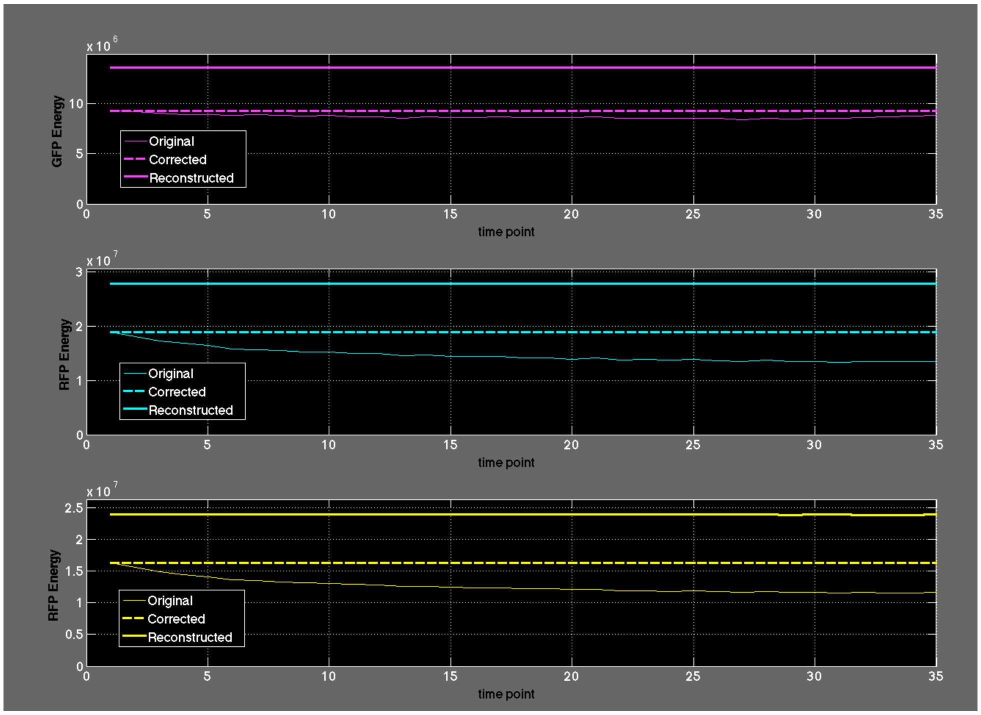

<u>Supplementary Figure 1.</u> Linear compensation of photo bleaching for three independent channels. After the numerical correction the fluorescence levels remain as a constant over time. 3-D line Interpolation is applied on the original 3D stack to compensate the anisotropic image acquisition, i.e. z-step is bigger than the x-y resolution.

Supplementary Fig. 2

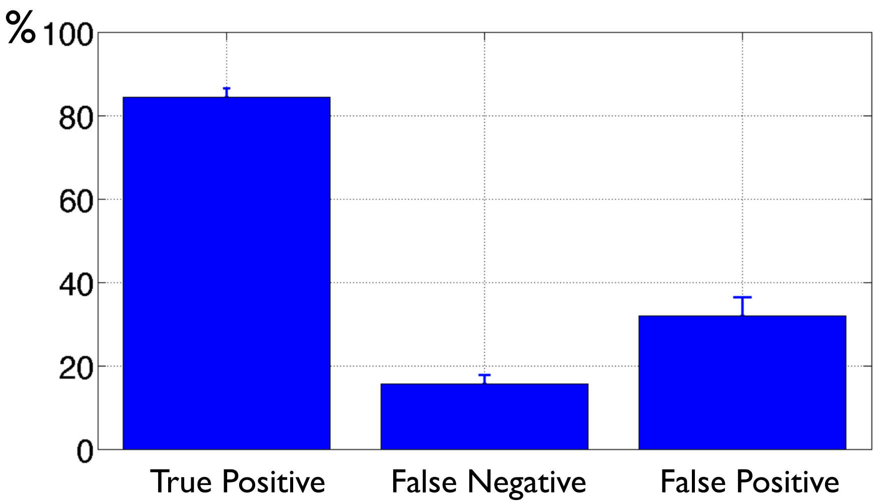

(Cell #=10)

<u>Supplementary Figure 2.</u> Comparison of the accuracy between computational segmentation results and manually annotated results. Let $\omega_j^g \;\; j=1,2,..N$ be the manual truth segments and $\omega_i^s \;\; i=1,2,..M$ be the computational segments. We calculate the true positive for each computational segment ω_i^s as $TP_i = \max_j \left(\frac{|\omega_i^s \cap \omega_j^g|}{|\omega_i^s \cup \omega_j^g|} \right)$. Where $|\cdot|$ means to calculate the area. The false positive is defined as $FP_i = \min_j \left(\frac{|\omega_i^s - \omega_i^s \cap \omega_j^g|}{|\omega_i^s \cup \omega_j^g|} \right)$ and the false negative is given as $FN_i = \min_j \left(\frac{|\omega_j^g - \omega_i^s \cap \omega_j^g|}{|\omega_i^s \cup \omega_j^g|} \right)$. Error bar represents the Standard Error of the mean.

Supplementary Fig. 3

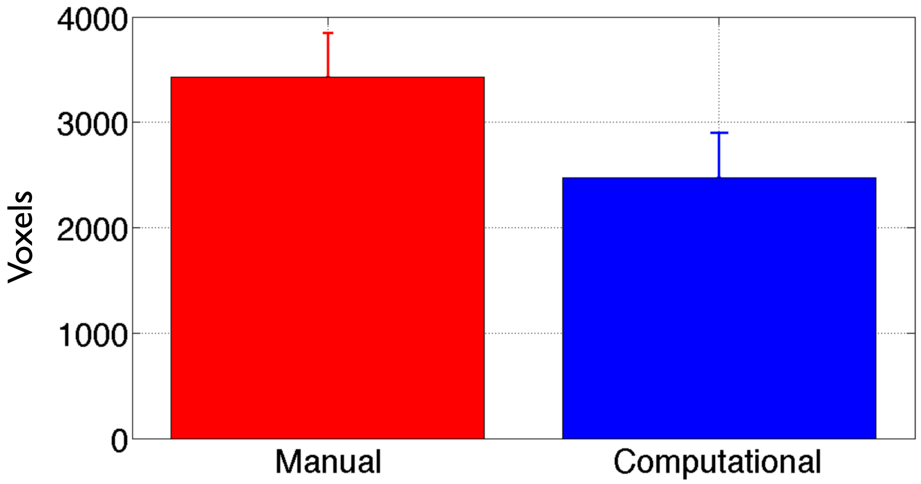

(Cell # = 10)

<u>Supplementary Figure 3.</u> Comparison of standard deviation of volume of computational segmentation and manual segmentation. Error bar represents the Standard Error of the mean. The computational results have smaller variation than the manual annotation.

Supplementary Fig. 4

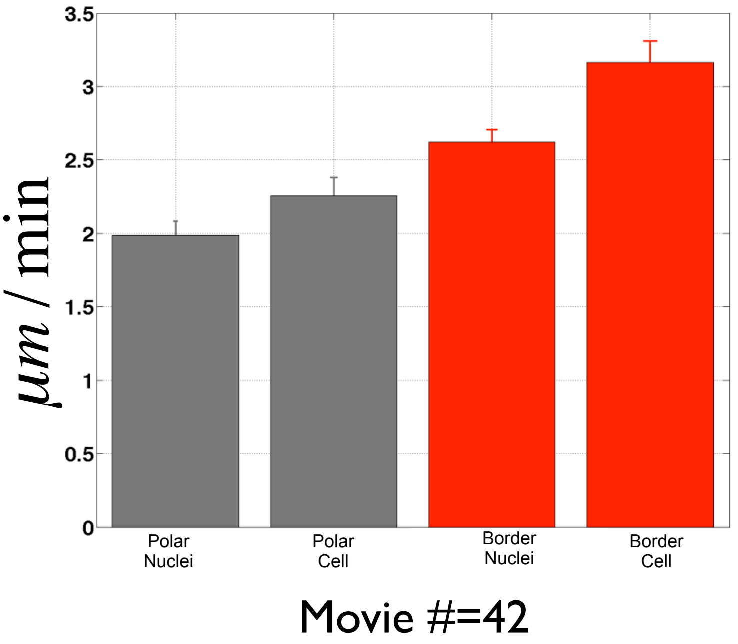

<u>Supplementary Figure 4.</u> Nucleus and cell speed magnitude for both polar and border cells. Error bar represents the Standard Error of the mean. In general, the nuclei speed is smaller than the cell and border cell moves faster than the polar cell.

Supplementary Fig. 5

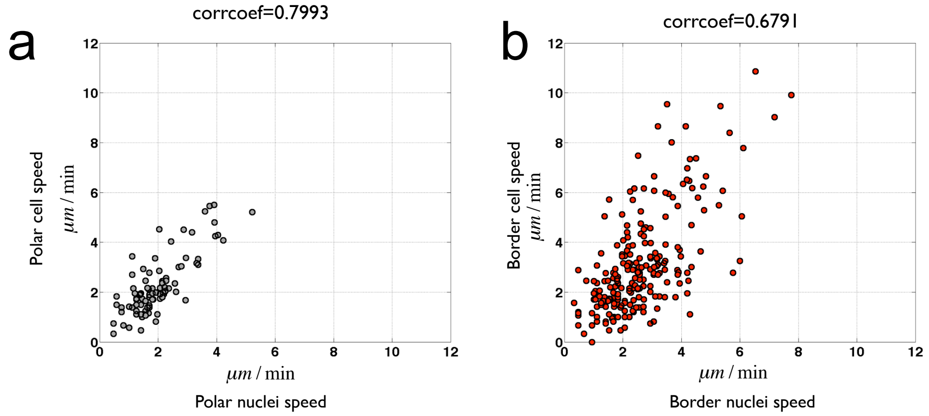

Supplementary Figure 5. Correlation between the nucleus and cell speed. (a) Correlation of polar nucleus speed and polar cell speed. (b) Correlation between the border nucleus speed and the border cell speed. The correlation coefficient is around 0.7, which indicates that the nucleus speeds and cell speeds are strongly correlated.

Supplementary Fig. 6

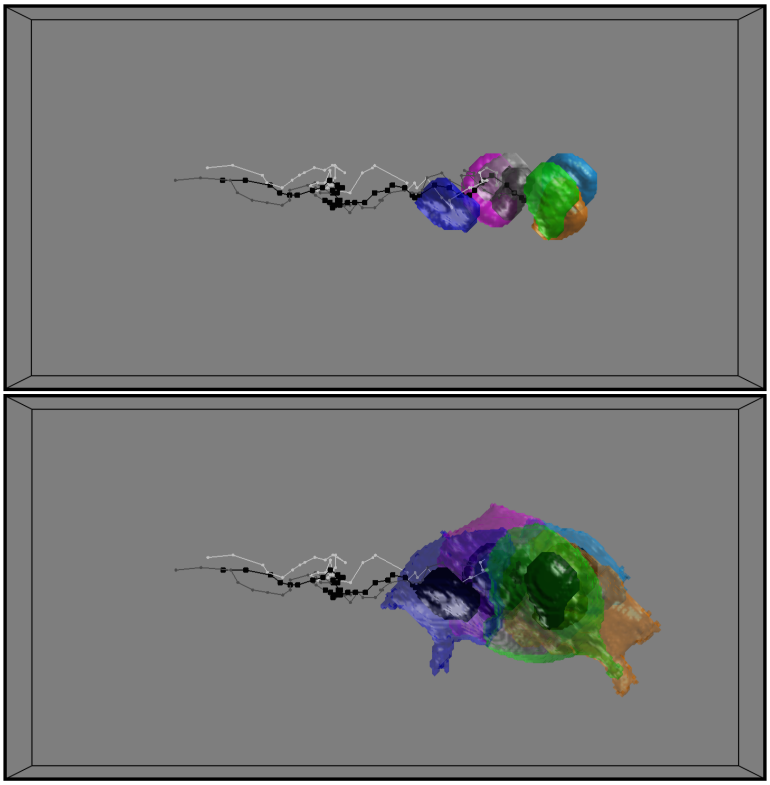

<u>Supplementary Figure 6.</u> Cluster mass center moving direction and the polar cells/nuclei trajectories. The polar cells/nuclei trajectories are indicated by light and dark gray lines. Their movements are very close to the cluster mass center, which is indicated by the black line.

Supplementary Fig. 7

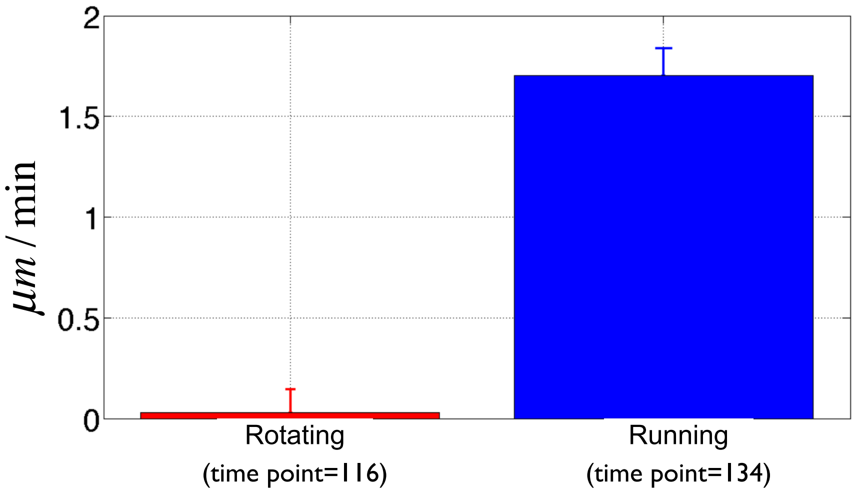

Supplementary Figure 7. The cluster net forward speed magnitude of rotating and running modes. Error bar represents the Standard Error of the mean. This confirmed that cluster does not move effectively during rotating mode, while in running mode is moves faster. (The velocity along the z direction is not considered.)

Supplementary Fig. 8

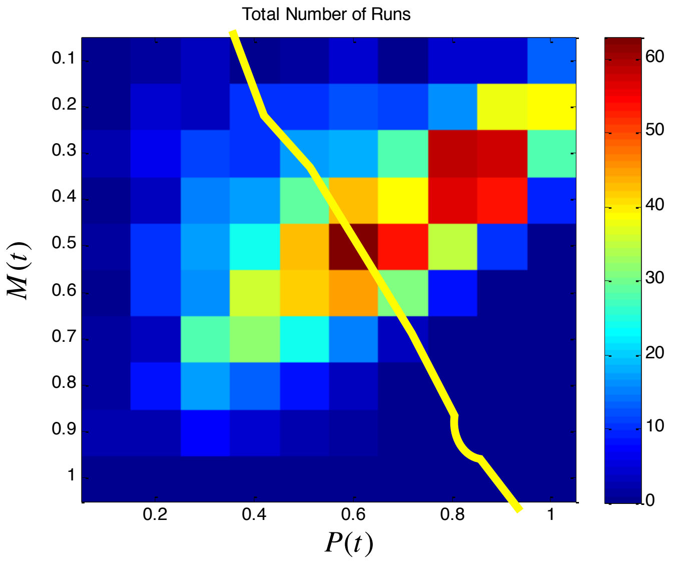

Movie #=42

<u>Supplementary Figure 8.</u> Distribution of Group Polarization and Angular Momentum over 42 movies. The cluster migration is generally directed and well coordinated. Yellow line is the decision boundary between clusters only with running and rotating as demonstrated in **Fig. 3d**. The random movement of the cells in the cluster will have low value for both GP and AM, while we observed that there is few time points at which both GP and AM is low (top-left corner).

Supplementary Fig. 9

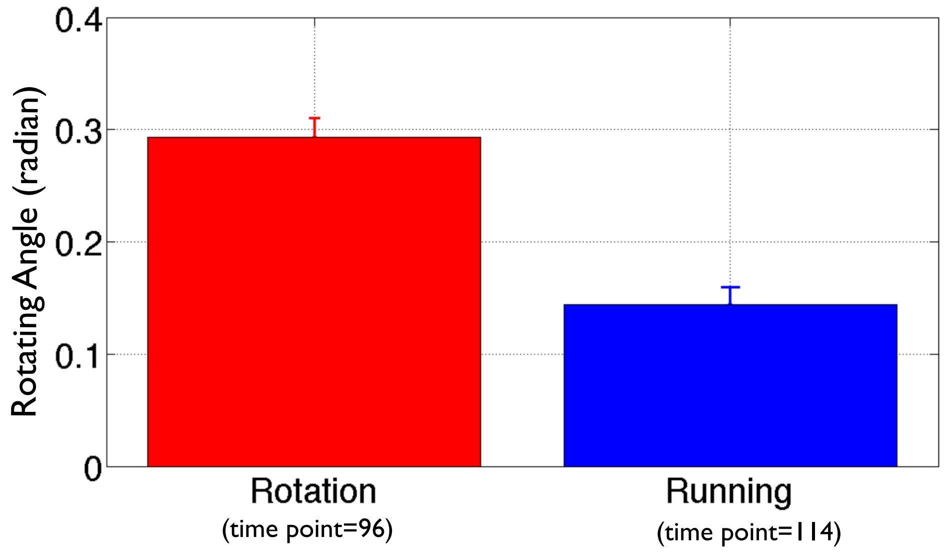

Supplementary Figure 9. Moving average of polar cell axis rotating angles in radius (over 5 time point) between rotating and running modes. Error bar represents the Standard Error of the mean. During the directed running mode, the polar axis will still vibrate a little bit, while the turning angle is much bigger during the rotating mode.

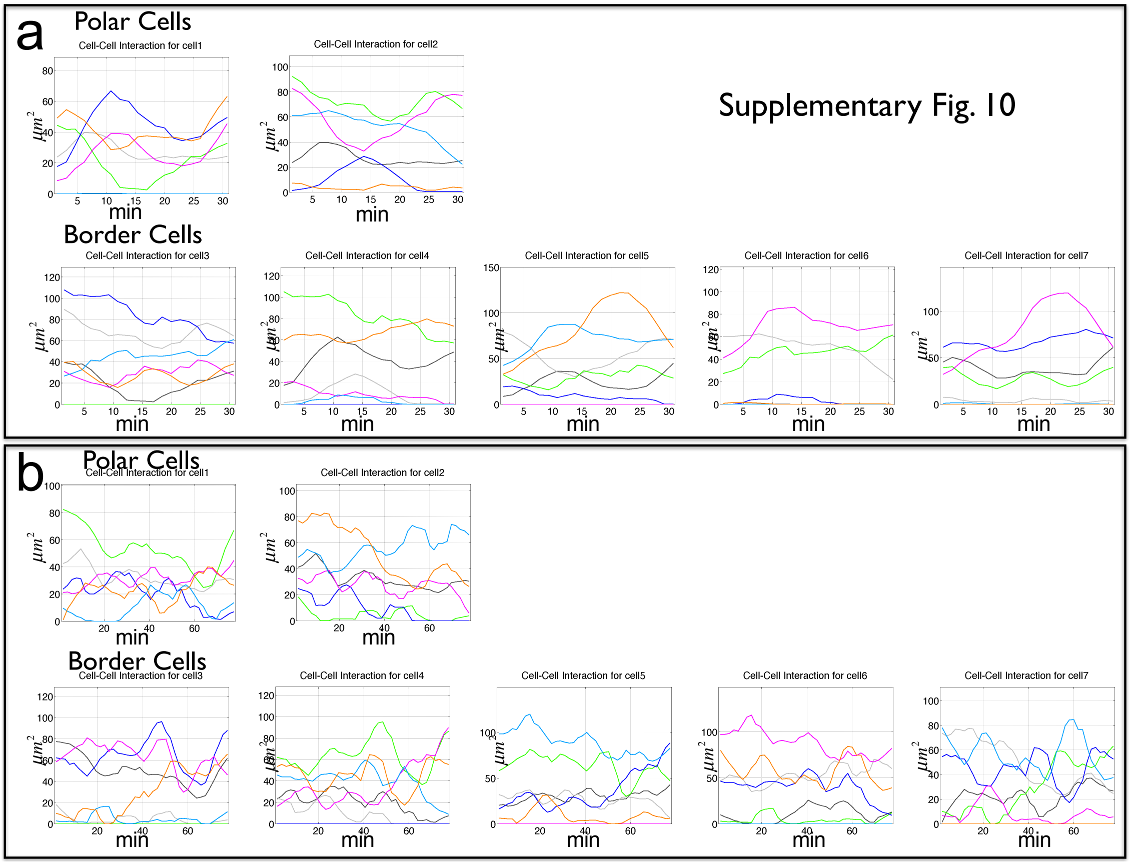

<u>Supplementary Figure 10.</u> Representative data for cell-cell interacting interface during running mode (a) and rotating mode (b). Different color indicates the cell identities. Based on visual inspection, our hypotheses are the interacting surfaces at the running mode is more stable and the variation of the interacting interface is bigger. This parameter might be relevant with the coordination of the cell during different modes and further investigation is needed.

Supplementary Fig. 11

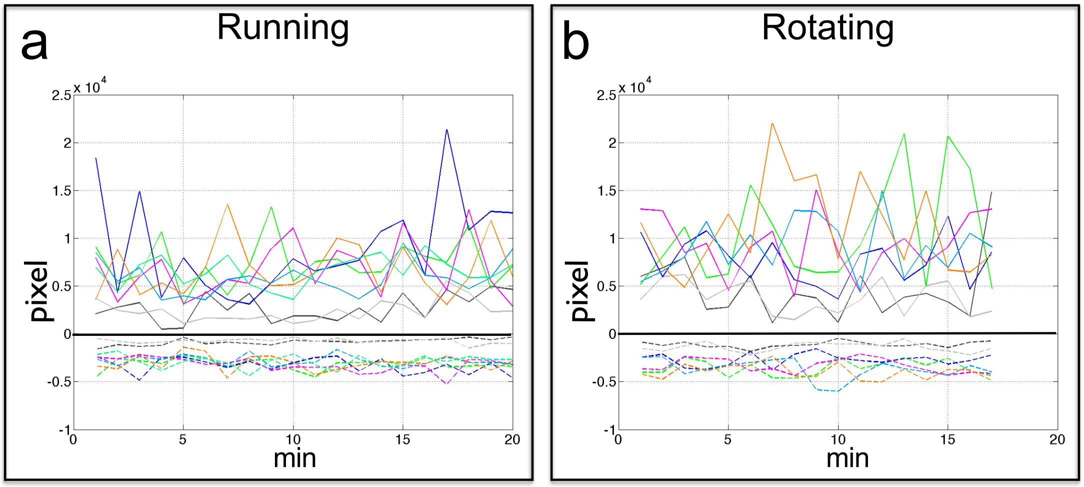

<u>Supplementary Figure 11.</u> The plot of Positive Deformation Energy (**PDE**) in solid lines and Negative Deformation Energy (**NDE**) in dashed lines for running and rotating. In general, the front edge of the cell is much more active then the rear edge.

Supplementary Fig. 12

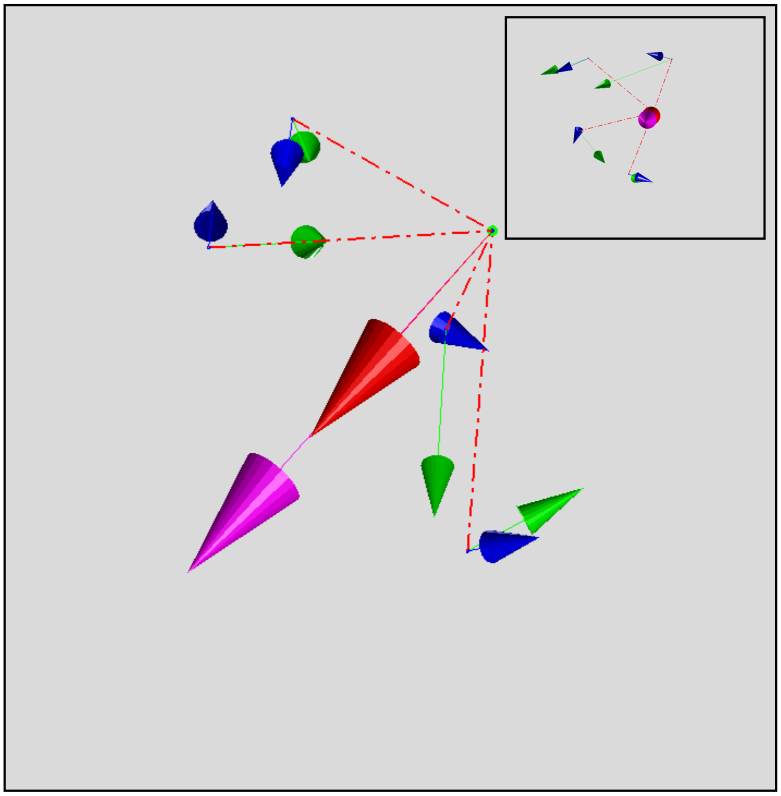

Supplementary Figure 12. The rotating movement of the cluster takes in consideration of all cell movements (represented by the nuclei center displacement vector) indicated by the blue arrow. The direction of a given cell to the cluster center is indicated by the red dashed line. The cross product of these two vectors gives a perpendicular vector and the combination of all those perpendicular vectors of all cells produces a combined cluster rotating vector indicated by the red arrow. Similarly, the cell positive deformation represents the cluster internal protrusion, illustrated by the green. Applying the same computation to those vectors, we can get a combined internal protrusion rotating vector, represented by the magnet arrow.
